# Zonation of bacterioplankton communities along aging upwelled water in the northern Benguela upwelling

**DOI:** 10.3389/fmicb.2015.00621

**Published:** 2015-06-18

**Authors:** Benjamin Bergen, Daniel P. R. Herlemann, Klaus Jürgens

**Affiliations:** Leibniz Institute for Baltic Sea Research WarnemündeRostock, Germany

**Keywords:** Benguela upwelling, diversity, pyrosequencing, bacterioplankton communities, phytoplankton bloom

## Abstract

Upwelling areas are shaped by enhanced primary production in surface waters, accompanied by a well-investigated planktonic succession. Although bacteria play an important role in biogeochemical cycles of upwelling systems, little is known about bacterial community composition and its development during upwelling events. The aim of this study was to investigate the succession of bacterial assemblages in aging upwelled water of the Benguela upwelling from coastal to offshore sites. Water from the upper mixed layer at 12 stations was sampled along two transects from the origin of the upwelling to a distance of 220 km. 16S rRNA gene amplicon sequencing was then used in a bacterial diversity analysis and major bacterial taxa were quantified by catalyzed reporter deposition-fluorescence *in situ* hybridization. Additionally, bacterial cell numbers and bacterial production were assessed. Community statistical analysis revealed a reproducible zonation along the two transects, with four clusters of significantly different microbial assemblages. Clustering was mainly driven by phytoplankton composition and abundance. Similar to the temporal succession that occurs during phytoplankton blooms in temperate coastal waters, operational taxonomic units (OTUs) affiliated with Bacteroidetes and Gammaproteobacteria were dominant during algal blooming whereas “Pelagibacterales” were highly abundant in regions with low algal abundance. The most dominant heterotrophic OTU (9% of all reads) was affiliated with “Pelagibacterales” and showed a strong negative correlation with phytoplankton. By contrast, the second most abundant heterotrophic OTU (6% of all reads) was affiliated with the phylum Verrucomicrobia and correlated positively with phytoplankton. Together with the close relation of bacterial production and phytoplankton abundance, our results showed that bacterial community dynamics is strongly driven by the development and composition of the phytoplankton community.

## Introduction

Ocean margins and upwelling systems in particular are sites of enhanced primary production and organic matter export. The Benguela upwelling, one of the most intensive upwelling systems worldwide (Nelson and Hutchings, 1983), is characterized by constant upwelling of nutrient rich water from deeper layers with maximum upwelling between August and October and a minimum between January and March (Shannon and Nelson, 1996). Upwelling is driven by southeast trade winds and the resulting Ekman offshore transport. Many studies on the Benguela upwelling have focused on the succession of phytoplankton and zooplankton. Their results have shown that the upwelling of nutrients leads to a characteristic phytoplankton succession in which the early bloom is dominated by diatoms or dinoflagellates (e.g., Barlow, 1982; Pitcher et al., 1998) that, after nutrient depletion, are replaced by other phytoplankton groups (Brown and Hutchings, 1987). As the bloom declines, there is an increase in nano- and mesozooplankton biomass and therefore in the grazing impact of flagellates and copepods (Painting, 1993a).

Investigations of the role of prokaryotes in upwelling systems have shown a significant correlation between bacterial and primary production (e.g., Painting, 1993b; Wiebinga et al., 1997; Cuevas et al., 2004) which indicates the importance of bacteria as decomposers of organic matter in these systems. Most studies have focused on prokaryotic bulk parameters such as cell abundance or production while the few that have examined upwelling-induced changes in prokaryotic community composition either lacked sufficient resolution (Kerkhof et al., 1999; Suzuki et al., 2001; Alonso-Gutiérrez et al., 2009; Teira et al., 2009b), were limited to one sampling station, or focused on metabolic processes (Cury et al., 2011; Zeigler Allen et al., 2012). Nonetheless, their findings provided the first clues in upwelling systems of the frequent occurrence of *Bacteroidetes*, *Roseobacter*, and the gammaproteobacterial clade SAR86 as well as specific associations between bacteria and phytoplankton.

As a perennial upwelling system, the northern Benguela provides optimal conditions to investigate bacterial community development along aging upwelled water during the successive stages of a phytoplankton bloom. Hansen et al. (2014) analyzed samples taken in parallel to those collected for this study and found a shift in phytoplankton community composition, with dinoflagellates dominating coastal stations and diatoms dominating the phytoplankton community located approximately 50 km offshore.

The present study was part of an interdisciplinary research project examining successive processes in the coastal Benguela upwelling system during strong upwelling in late winter. Applying an Eulerian approach, we sampled a transect at a 45° angle to the upwelling current, which led to a projection of aging upwelled water along the transect (Mohrholz et al., 2014). To determine whether, as expected, the bacterial community responds to upwelled water of different ages, we used next-generation sequencing of partial 16S rRNA genes to describe the changes in community composition. The data were analyzed for indications of the major drivers of changes in the bacterial community. This study is the first to demonstrate the remarkable differences in the bacterial communities of the Benguela upwelling and that they are mainly triggered by the abundance and quality of phytoplankton. On a spatial scale, our findings also confirm the successional bacterial pattern that occur during phytoplankton blooms.

## Materials and Methods

### Sampling

**FIGURE 1:**
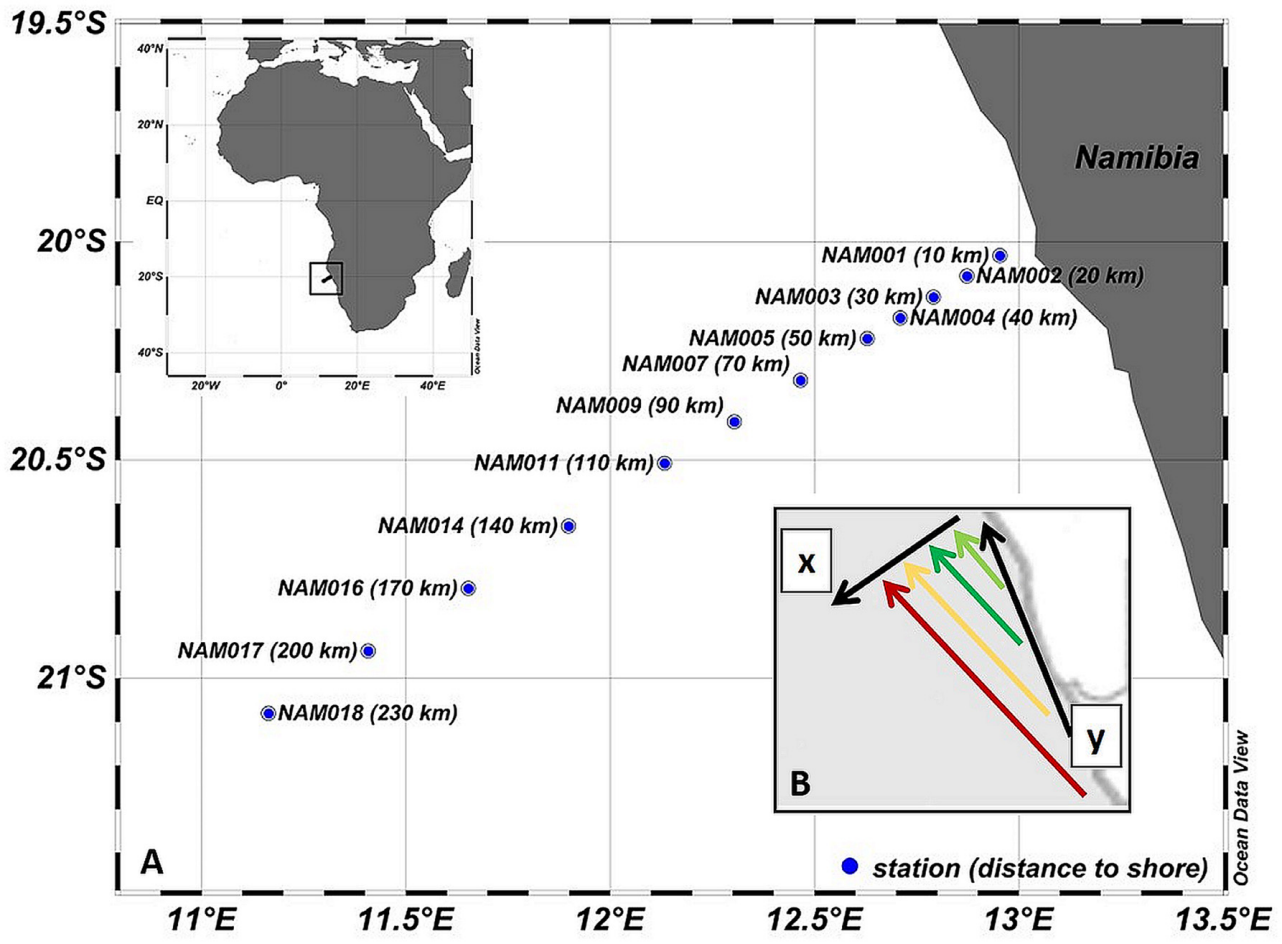
**(A)** Transect along the northern Benguela upwelling, showing the locations of the stations and their distance to the shore. **(B)** Scheme of the Eulerian approach used in this study, with the coastal parallel current vector y and the normal to the coast vector x that corresponds to the transect. The colored vectors indicate the projection of the upwelled water during different developmental stages on the transect.

Samples were taken between 10 and 220 km off the coast of Namibia during a cruise of the R/V M. S. Merian in the northern Benguela upwelling region (**Figure 1**). The transect was sampled two times consecutively with a time interval of 4 days (transect 1: 27.08.2011–30.08.2011; transect 2: 30.08.2011–02.09.2011). Samples were taken at depths of 5 and 20 m using a rosette water sampler comprising 24 10-L free-flow bottles. Profiles of temperature, salinity, oxygen, and chlorophyll fluorescence were measured using a CTD SBE911+ combined with the bottle sampler rosette. Water samples for DNA analysis were filtered onto 0.22-μm pore-size white polycarbonate filters. DNA was extracted according to Weinbauer et al. (2002). Chlorophyll *a* (Chl-a) concentrations were determined according to Hansen et al. (2014).

### Prokaryotic Cell Number and Activity Measurements

Prokaryotic cells were counted using a flow cytometer (FacsCalibur, Becton Dickinson, Heidelberg, Germany) following the method of Gasol et al. (1999). Calculations were performed using the software program “Cell Quest Pro,” plotting the emission fluorescence of SYBR Green I (488 nm) vs. the side scatter. Picocyanobacteria were similarly counted on the basis of their signature in a plot of orange (FL2) vs. red (FL3) fluorescence.

The incorporation of ^3^H-leucine (140 Ci mmol^-1^) was measured to estimate heterotrophic bacterial productivity in 10-mL water samples. Triplicate samples were incubated at a final concentration of 100 nM for at least 1 h at the *in situ* temperature in the dark. Incorporation was stopped by fixing the cells with formaldehyde (5% v/v). A fourth sample, serving as a blank, was fixed for at least 10 min prior to the addition of the radioactively labeled substrate. The samples were filtered onto 0.22-μm polycarbonate filters (Millipore), which were then placed in 4 mL of scintillation cocktail. The incorporated substrate was counted in a scintillation counter (Packard). Bacterial carbon production was calculated from ^3^H-leucine incorporation according to Simon and Azam (1987), using a leucine mol% value of 7.3 and a carbon conversion factor of 0.86.

### Catalyzed Reporter Deposition-Fluorescence *in Situ* Hybridization (CARD-FISH) and Cell Counting

Catalyzed reporter deposition-fluorescence *in situ* hybridization was carried out using the protocol of Pernthaler et al. (2002), with modifications. Before digestion, the filters were incubated in 0.01 M HCl for 10 min to inactivate undesirable peptidases. Bacterial staining was carried out using the horseradish-peroxidase-labeled FISH probes EUBI-III (Daims et al., 1999), VER47 (Buckley and Schmidt, 2001), and SAR11-486 (Fuchs et al., 2005). For signal amplification, tyramide labeled with the fluorescent dye carboxyfluoresceine was used. Total cell numbers were estimated by 4′,6′-diamidino-2-phenylindole (DAPI)- staining of the probe-labeled samples.

DAPI and EUB I-III stained cells were counted using an automated system coordinated with the epifluorescence microscope AxioImager (Zeiss, Germany) and in combination with a Colibri LED unit and a charge-coupled device camera (AxioCam MRm, Zeiss, Germany). Images were acquired using a 100× Plan-Apochromat objective (Zeiss) and the Zeiss multi-band filter set 62HE. Automatic processing of the samples was achieved with the Visual Basic for Application module of AxioVision 4.6 (Zeiss, Germany) together with comprised automated sample recognition and localization, multichannel image acquisition, image processing, and cell counting routines (Zeder and Pernthaler, 2009). VER47- and SAR11-486 stained cells were counted manually at the same microscope using a 63× Plan-Apochromat objective and the same filter set. For each sample, at least 1000 DAPI-stained cells in at least ten independent microscopic fields were counted, excluding cells that exhibited autofluorescence (590 nm). Heterotrophic nanoflagellates (HNFs) were also counted manually using filter set 02 (Zeiss, Germany). A minimum of 100 cells per filter were counted at a magnification of 630×.

### Bacterial Community Composition

For bacterial diversity analysis, hypervariable regions 3–5 (V3–V5) of the 16S rRNA gene were used to generate PCR amplicons, as described by Herlemann et al. (2011), but with a modification of 30 PCR cycles. Sequencing was performed by Eurofins MWG GmbH using 454 GS-FLX sequencer (Roche). The denoising tool Acacia (Bragg et al., 2012) was used to correct amplicon pyrosequencing errors. Primer sequences were trimmed from the reads and the sequences were clipped 400 bp downstream of the primer. Reads shorter than 400 bp (excluding the primer) and/or containing Ns were excluded. Reads that were found only once in the sample set were removed from the analysis. Sequences were aligned and clustered at 97% identity into operational taxonomic units (OTUs), as described by Herlemann et al. (2011), using the pyrosequencing pipeline at RDP (Cole et al., 2009). The online tool Decipher (Wright et al., 2012) was used to identify and remove chimeric sequences in the remaining OTUs. The abundances of the resulting OTUs were normalized using the relative proportions of individual OTU reads from all sample reads. Sequences have been deposited in the European Nucleotide Archive (ENA), with the study accession: http://www.ebi.ac.uk/ena/data/view/PRJEB8816.

### Statistics

A non-hierarchical clustering method, *k*-means clustering (MacQueen, 1967), was used to reveal similar spatial distribution patterns in the relative abundances of the 25 most frequent OTUs. According to the most pronounced distribution patterns, OTUs were clustered into five groups. Sequence results from the whole sequence data were analyzed using principal coordinate analysis (PCoA) with the Bray–Curtis index of dissimilarity based on the normalized abundance data. A cluster analysis was used to identify bacterial community clusters in the PCoA plot and a subsequent ANOSIM analysis was performed to confirm significant differences between the clusters. Spearman rank correlation analysis was used to compare PCoA coordinates with environmental parameters to determine their link to community separation. All statistical analysis were performed using the PAST software package version 2.17c (Hammer et al., 2001).

## Results

Hydrographic data showed that this study was performed during a period of constant upwelling and the offshore transport of nutrient-rich shelf water in front of Namibia (Mohrholz et al., 2014; Nausch and Nausch, 2014). Since the mixed-layer depth was at least 20 m along the whole transect, for all analyses in this study the mean values from the 5 and 20-m samples were used to describe bacterial community development in the upper mixed layer. Upwelling resulted in an increase in phytoplankton development, characterized by a high abundance of dinoflagellates, beginning 40 km offshore, at station NAM004, and peaking 70 km offshore, at station NAM007, where diatoms dominated (Hansen et al., 2014). A cold-water filament moving offshore influenced the remote stations (NAM016 to NAM018), causing minor differences between transects 1 and 2, namely, a more pronounced Chl-a peak at station NAM007 in transect 2 and a shift of the offshore Chl-a filament from station NAM017 in transect 1 to station NAM018 in transect 2. Therefore, the developmental stage of the upwelled water was classified to determine the validity of a description of successive processes along the transect or whether it would be affected by mesoscale dynamics such as filaments and eddies. The progressive aging of the water masses from station NAM001 to NAM014 was confirmed by Mohrholz et al. (2014), who used salinity, temperature, and oxygen concentrations to calculate the relative ages (pseudoages) of the water masses.

### Prokaryotic Abundance and Activity

**FIGURE 2:**
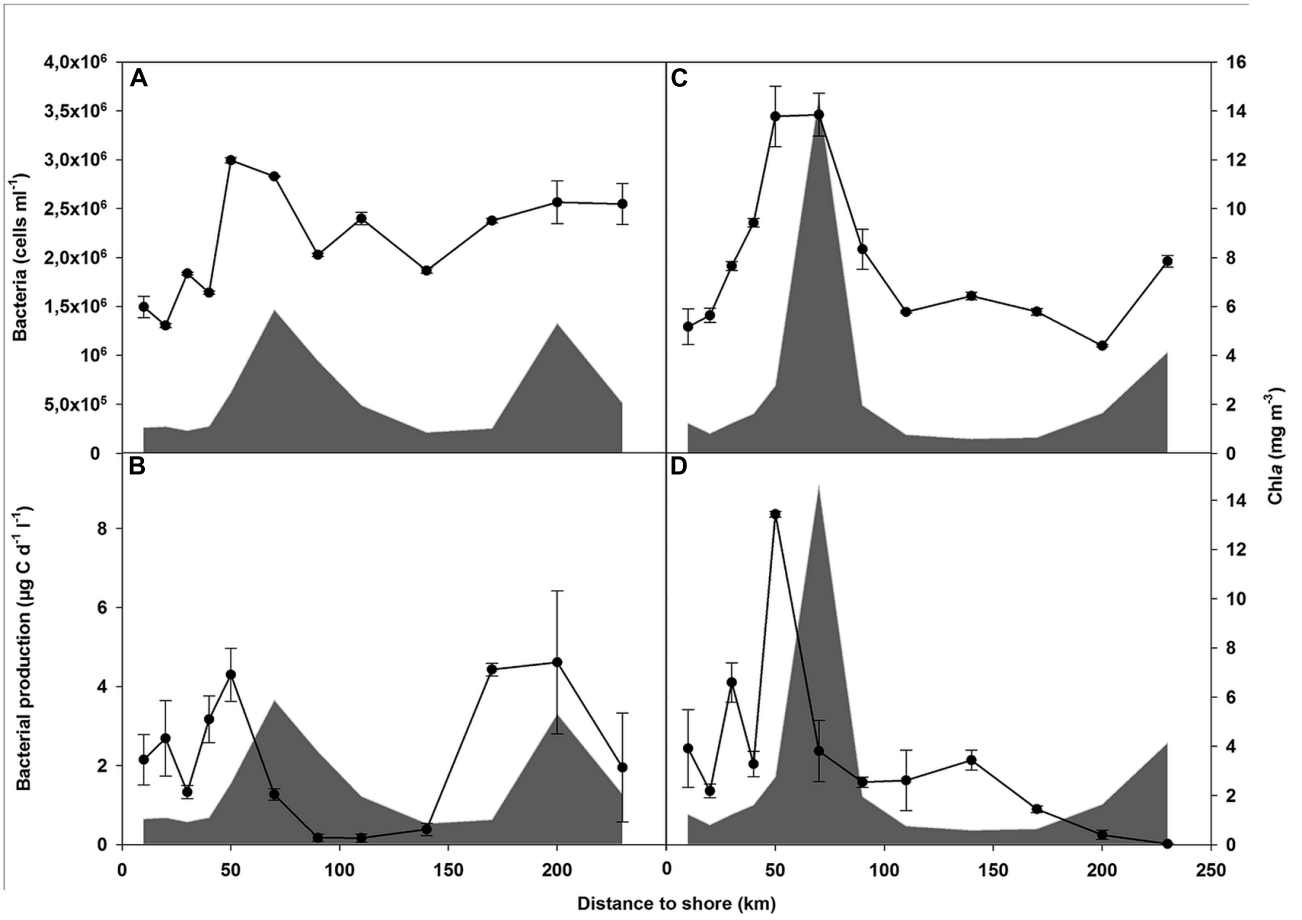
**Prokaryotic abundance (PA) in transect 1 **(A)** and transect 2 **(C)** and bacterial protein production (BPP) in transect 1 **(B)** and transect 2 **(D)** in the upper 20 m (lines).** Chlorophyll-a concentration (gray area) in relation to the distance from the coast is also shown.

Prokaryotic abundance (PA) ranged from 1.3 × 10^6^ cells mL^-1^ at the near-shore station (NAM001, transect 2) to a peak of 3.5 × 10^6^ cells mL^-1^ at a distance of 70 km from the coast (NAM007, transect 2; **Figures 2A,C**). The increase in PA over the first 70 km was followed by a decrease until 90 km, remaining relatively constant, at 1.5–2 × 10^6^ cell mL^-1^, thereafter. PA correlated significantly with Chl-a concentrations (*p* < 0.05). As most stations with high Chl-a concentrations were dominated by diatoms (Hansen et al., 2014), there was a significant positive correlation between PA and diatom biomass (*p* < 0.05). Further analysis showed significant positive correlations between PA and NO_2_ (*p* < 0.05) and a marginally significant correlation with the abundance of HNFs (*p* < 0.1; **Table 1**).

**Table 1:** Correlation (Spearman, *r*_s_) of bacterial parameters and environmental variables from both transects.

Parameter	PA*r*-, *p*-value	BPP*r*-, *p*-value
NO_2_	0.49, <0.05	ns
NO_3_	ns	0.47, <0.05
PO_4_	ns	0.52, <0.05
NH_4_	ns	ns
Chl-a	0.71, <0.05	ns
Diatom^a^	0.57, <0.05	ns
Dino^a^	ns	ns
HNF	ns	ns

*^a^Expressed as biomass*.

*PA, prokaryotic abundance; BPP, bacterial protein production; Chl-a, chlorophyll-a; Dino, dinoflagellates; HNF, heterotrophic nanoflagellate cell number; ns, not significant*.

*Synechococcus* cell numbers ranged from 6.5 × 10^6^ cells L^-1^ at the near-shore stations of transect 1 to 5.8 × 10^7^ cells L^-1^ in the region of low Chl-a concentration of transect 2 (NAM014). The *Synechococcus* distribution pattern was in good accordance with bacterial protein production (BPP) but the trend was not significant (data not shown). *Prochlorococcus* cells were not detected in either transect.

Bacterial protein production increased in parallel with PA over the first 50 km, from 2.3 μg C d^-1^ L^-1^ ± 0.2 μg C d^-1^ L^-1^ at the most near-shore station (NAM001; means of transect 1 and 2) to 6.33 μg C d^-1^ L^-1^ ± 2.88 μg C d^-1^ L^-1^ at station NAM005 (**Figures 2B,D**). However, the peak in BPP occurred before the peak in Chl-a concentration in areas with high nutrient levels. At a distance of 70 km (NAM007), where PA was high and Chl-a concentrations reached a maximum, BPP strongly decreased to 1.82 μg C d^-1^ L^-1^ ± 0.78 μg C d^-1^ L^-1^. A second but smaller increase in BPP, to 2.67 μg C d^-1^ L^-1^ ± 2.5 μg C d^-1^ L^-1^, was measured at transect 1 at a distance of 170 km (NAM016). Bacterial production was positively correlated to nutrients (NO_3_, PO_4_, DIN; *p* < 0.05) and was marginally significant correlated with the abundance of HNF (*p* < 0.1; **Table 1**).

### Bacterial Diversity

Pyrosequencing generated 150,006 raw sequence reads. After denoising and the removal of sequence reads present only once in all samples and of chimeric sequences, a total of 125627 reads remained. These sequences were clustered into 1335 OTUs at an average sequence identity of 97% per OTU.

Analysis of the normalized abundance data showed that the 25 most abundant OTUs contained more than 50% of all reads and were identical in both transects, although in a different rank order (**Table 2**). The most abundant OTUs in both transects were those of common marine bacterial groups, including two OTUs affiliated with “Pelagibacterales” (10% of the total read abundance), one OTU from *Cyanobacteria* family II (9.5%), which includes the genera *Prochlorococcus* and *Synechococcus*, one OTU identified as *Verrucomicrobia* genus *Persicirhabdus* (5.4%), and two OTUs from the *Gammaproteobacteria* SAR86 cluster (4.9%; Supplementary Figures S1 and S2). The highest diversity occurred within the *Flavobacteriaceae*, represented by eight of the 25 most abundant OTUs.

**Table 2:** Taxonomic affiliation of the 25 most abundant operational taxonomic units (OTUs) along transect 1 and transect 2.

OTU no.	OTU	Transect 1 (rank)	Transect 1 (%)	Transect 2 (rank)	Transect 2 (%)
1	Cyanobacteria – *Synechococcus*	2	8.2	1	10.8
2	Alphaproteobacteria – *Candidatus Pelagibacter*	1	8.3	2	9.8
3	Verrucomicrobia – *Persicirhabdus*	3	5.3	3	5.5
4	Gammaproteobacteria – SAR86 clade	4	4.1	4	3.4
5	Gammaproteobacteria – *Oceanospirillales*	5	3.5	6	2.3
6	Actinobacteria – OCS155 marine group	7	2.5	5	2.6
7	Bacteroidetes – Flavobacteriaceae	6	2.6	8	2.2
8	Gammaproteobacteria – OM60(NOR5) clade	8	2.2	7	2.2
9	Alphaproteobacteria – Rhodobacteraceae	9	2.2	9	1.9
10	Bacteroidetes – Formosa	13	1.3	10	1.9
11	Alphaproteobacteria – *Roseobacter clade* DC5-80-3	10	1.7	13	1.4
12	Bacteroidetes – Flavobacteriaceae	12	1.5	11	1.4
13	Alphaproteobacteria – Rhodobacteraceae	11	1.7	20	1.0
14	Bacteroidetes – Formosa	14	1.3	12	1.4
15	Bacteroidetes – NS4 marine group	15	1.3	16	1.2
16	Gammaproteobacteria – E01-9C-26 marine group	18	1.1	17	1.2
17	Bacteroidetes – *Gaetbulibacter*	17	1.1	18	1.1
18	Gammaproteobacteria – SAR86 clade	16	1.2	21	1.0
19	Chloroplast	22	1.0	14	1.3
20	Bacteroidetes – NS2b marine group	19	1.1	15	1.2
21	Verrucomicrobia – *Roseibacillus*	23	0.9	19	1.1
22	Alphaproteobacteria – SAR11 clade	21	1.0	22	0.9
23	Bacteroidetes – VC2.1 Bac22	20	1.0	25	0.8
24	Bacteroidetes – *Ulvibacter*	24	0.9	23	0.8
25	Alphaproteobacteria – AEGEAN-169 marine group	25	0.8	24	0.8

*Abundance rank and averaged relative abundance of the respective OTUs are shown*.

### Spatial Patterns

**FIGURE 3:**
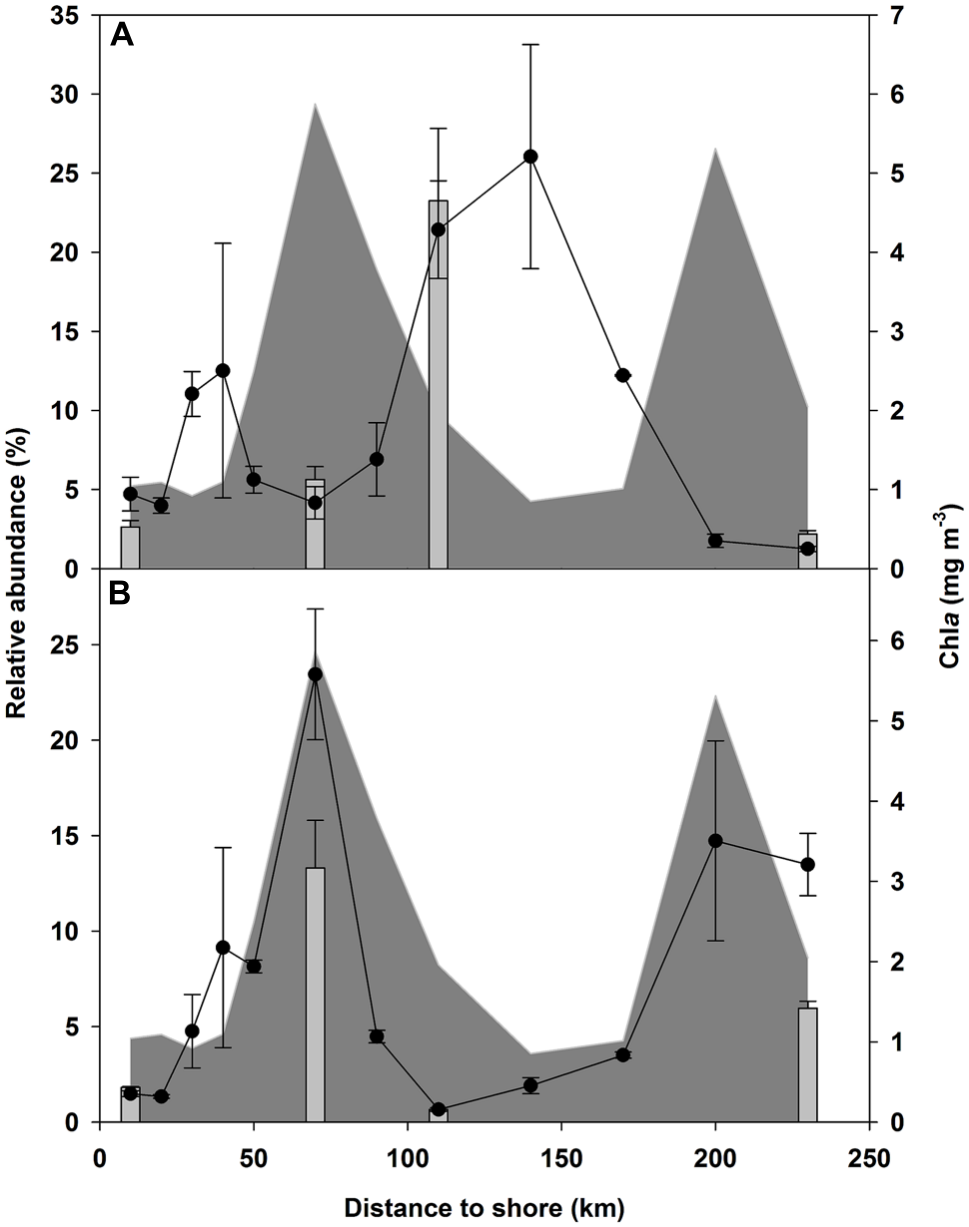
**Abundance profiles of “Pelagibacterales” **(A)** and *Verrucomicrobia***(B)** as the average of the upper 20 m of transect 1.** Dots indicate the relative amounts of the respective reads from all reads obtained from pyrosequencing, bars indicate the relative amounts of respective cells from all bacterial cells detected by Catalyzed reporter deposition-fluorescence *in situ* hybridization (CARD-FISH). The chlorophyll-a concentration is indicated by the gray area.

Some of the most abundant OTUs showed contrary patterns with respect to their relative abundances along the transects. For example, the abundances of OTUs affiliated with “Pelagibacterales” correlated negatively with those of *Verrucomicrobia* (*p* < 0.05). “Pelagibacterales” were dominant at stations with low Chl-a concentrations, but at stations with high Chl-a concentrations their relative abundances decreased whereas verrucomicrobial OTUs increased to as high as 23% of all bacterial reads (**Figure 3**), resulting in a significant positive correlation with Chl-a (*p* < 0.05). To validate the proportion of these abundant OTUs revealed by pyrosequencing, CARD-FISH was performed for a subset of eight samples from stations related to four clusters identified, as described below, by PCoA (5 and 20-m samples from NAM001, NAM007, NAM011, and NAM018). The proportions of “Pelagibacterales” and *Verrucomicrobia* cells from all bacteria calculated from the direct cell count using CARD-FISH showed similar trends to sequence proportions derived from pyrosequencing (**Figure 3**). However, for *Verrucomicrobia* the CARD-FISH-derived abundance was lower than that determined by pyrosequencing (CARD-FISH *n* = 8, average 5% of EUB, pyrosequencing average 10% of the total bacterial community) whereas for “Pelagibacterales” the abundances determined by the two methods were the same (CARD-FISH *n* = 8, average 8% of EUB, pyrosequencing average 8% of the total bacterial community). Linear regression analysis of the pyrosequencing- and CARD-FISH-derived abundances, including all samples, showed that their relationship was significant (*n* = 16, *R*^2^ = 0.77, *p* < 0.01).

**FIGURE 4:**
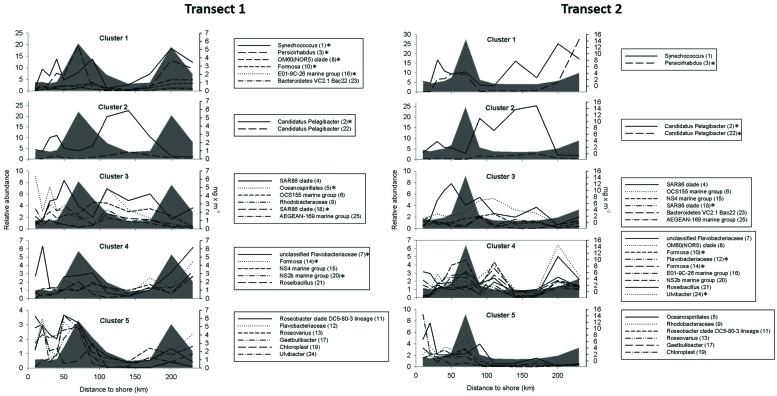
**Abundance profiles for the 25 most abundant operational taxonomic units (OTUs) along the two transects.** The OTUs were clustered according to their spatial distribution into five clusters by *k*-means clustering. The gray area indicates the chlorophyll-a concentration. The OTU ID is shown in parentheses to differentiate OTUs with identical taxonomic labels. Significant correlation with the chlorophyll-a concentration (^∗^*p* < 0.1).

*K*-means clustering was used to detect characteristic patterns of OTU abundance along the transects (**Figure 4**). The 25 most abundant OTUs could thus be assigned to five clusters with different patterns that were recognizable in both transects. The clustering approach mainly grouped OTUs according to their correlation with Chl-a. In both transects, cluster 1 and cluster 4 were mainly represented by OTUs that had a significant positive correlation with Chl-a (*p* < 0.1), including OTUs affiliated with *Synechococcus*, *Verrucomicrobia* genus *Persicirhabdus*, and several *Flavobacteriaceae*. Cluster 2 and cluster 3 comprised several OTUs that had a significant negative correlation with Chl-a, including “Pelagibacterales” and SAR86 clade OTUs. Cluster 5 contained OTUs that showed no correlation with Chl-a. Interestingly, cluster 4, which grouped OTUs that peaked in parallel during and after the bloom, was almost entirely represented in both transects by OTUs affiliated with *Flavobacteriaceae*.

**FIGURE 5:**
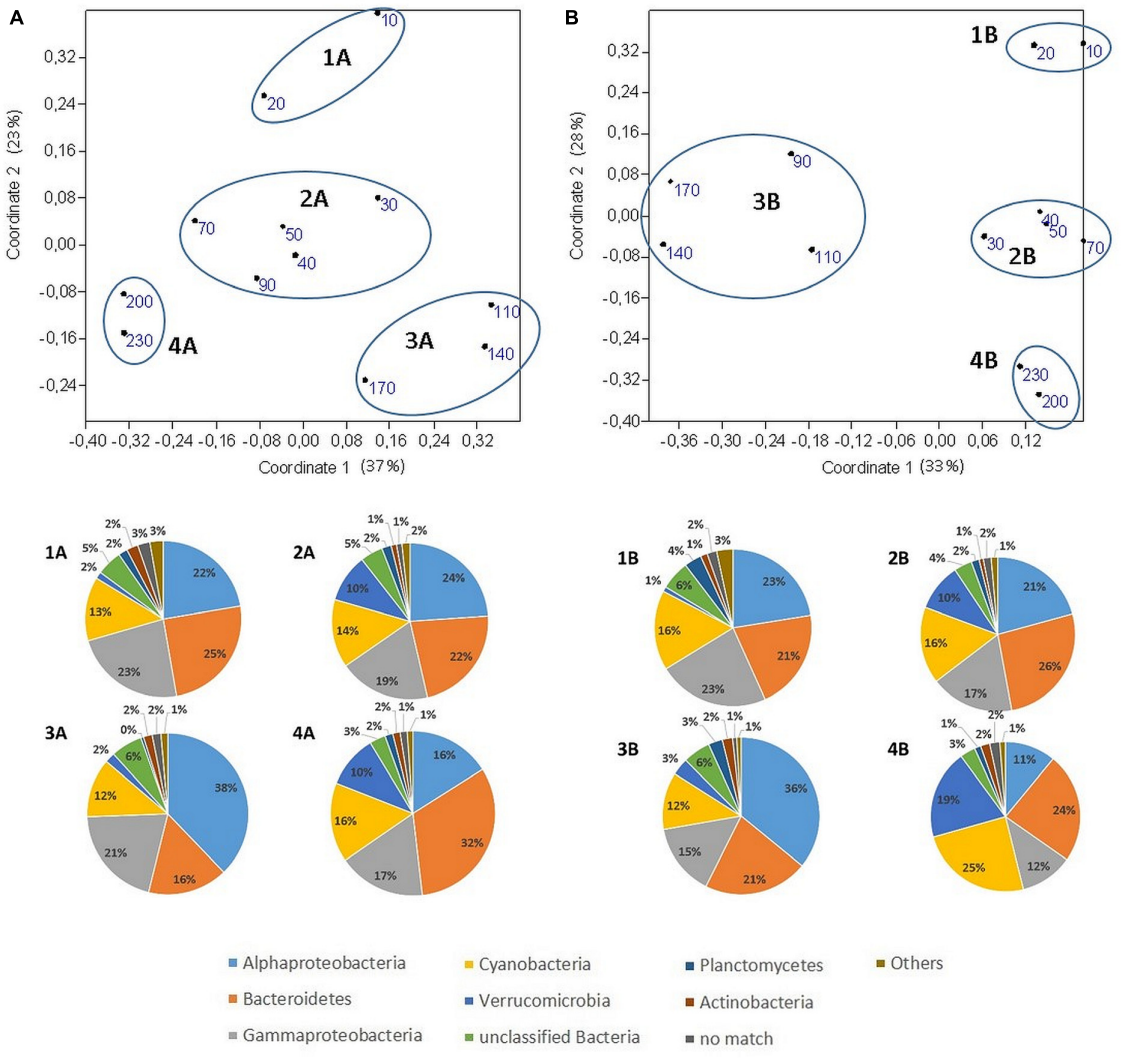
**Principle coordinate analysis (PCoA) of the normalized abundance of all OTUs based on the Bray–Curtis dissimilarity.** Each number corresponds to the distance to shore (in km) of the respective sample. The first three coordinates of the PCoA explained 71% of the variance (coordinate 1: 37%. 2: 23%. 3: 11%) in the community data of transect 1 **(A)** and 73% of the variance (coordinate 1: 33%. 2: 28%. 3: 12%) in the community data of transect 2 **(B)**. The pie charts show the relative abundances on the phylum level and refer to the respective clusters marked with blue ellipses in the PCoA plots. Stations were clustered according to the results of the Bray Curtis clustering analysis (see Supplementary Figure S2).

To identify a general structure in the distribution of OTUs along the transects, the relative abundances of all OTUs were subjected to a PCoA (**Figure 5**). Correlation analysis with environmental variables indicated that in both transects the first axis distinguished samples with low vs. high Chl-a values. The first axis divided samples from transect 2 by temperature and salinity (**Table 3**). In the second axis, samples from both transects were divided based on inorganic nutrient levels, temperature, and distance to shore. The analysis showed a zonation along the transects and a cluster analysis distinguished four bacterial community clusters defined at the OTU level (Supplementary Figure S3). An ANOSIM analysis confirmed the significant differences between these four clusters (transect 1: *R* = 0.83, *p* < 0.01; transect 2: *R* = 0.68, *p* < 0.01). The four clusters were numbered according to their distance to the shore, with increasing distance from cluster 1 to cluster 4. Their major features are presented in **Table 4**. Even on a phylum level, differences between the four clusters were obvious and they were apparent in both transects. Thus, all clusters comprised the same dominating phyla (*Alphaproteobacteria, Bacteroidetes, Gammaproteobacteria, Cyanobacteria,* and *Verrucomicrobia*) but their relative abundances differed greatly among clusters 1–4 (**Figure 5**). Whereas there was no clear trend in the proportion of OTUs affiliated with *Cyanobacteria*, the relative abundance of *Gammaproteobacteria* sequences was highest in the near-shore cluster 1 (23%) and lowest in the off-shore cluster 4 (14.5% ± 2.5%). *Alphaproteobacteria* reads were most abundant in cluster 3 (37% ± 1%) but very low in cluster 4 (13.5% ± 4%). By contrast, the proportion of sequences affiliated with *Bacteroidetes* was highest in cluster 4 (28% ± 2.5%) and lowest in cluster 3 (18.5% ± 2.5%). The strongest variations were observed in the relative abundances of *Verrucomicrobia* sequences, which were very low in clusters 1 and 3 (2% ± 0.8 %) but much higher in clusters 2 and 4 (12.25% ± 4.5%).

**Table 3:** Significant relationships between environmental parameters and principal coordinate analysis (PCoA) ordination of the normalized abundance of all OTUs for transect 1 and transect 2.

		Transect 1			Transect 2		
Coordinate	Variable	*n*	*r*_s_	*p*	*n*	*r*_s_	*p*
**1**							
	Chl-*a*	12	-0.76	<0.01	12	0.6	0.03
	Diatom biomass	9	-0.66	0.05			
	PO_4_				12	0.59	0.04
	Temperature				12	-0.63	0.03
	Salinity				12	-0.80	<0.01
	Pseudoage				12	-0.63	0.03
**2**							
	Salinity	12	-0.86	<0.01			
	Dinoflagellate biomass	9	-0.66	0.05			
	NO_3_	12	0.94	<0.01	12	0.62	0.03
	PO_4_	12	0.88	<0.01	12	0.63	0.03
	SiO_4_	12	0.92	<0.01	12	0.67	0.02
	DIN	12	0.94	<0.01	12	0.63	0.03
	Temperature	12	-0.97	<0.01	12	-0.62	0.03
	O_2_	12	-0.79	<0.01	12	-0.70	0.01
	Distance	12	-0.90	<0.01	12	-0.72	0.01
	Pseudoage	11	-0.95	<0.01	12	- 0.59	0.04

*The number of data (n), Spearman correlations (r_s_), and significance values (p) are shown. Chl-a, chlorophyll a; DIN, dissolved inorganic nitrogen*.

**Table 4:** Major phytoplankton groups and OTUs (according to the values shown in **Table 2**) in the four zones determined by PCoA and the respective diversity measures.

Zone	1	2	3	4
Major phytoplankton	Dinoflagellates	Dinoflagellates/Diatoms	Dinoflagellates	Diatoms
Major OTUs	5, 1, 19, 2, 7	1, 2, 3, 4, 5	2, 1, 4, 6, 5	1, 3, 8, 7, 14
Shannon (H)	9.235	7.082	8.256	6.04
Richness (S)	862	888	868	514

## Discussion

The northern Benguela is a highly productive, perennial upwelling system (Shannon et al., 1986). The aim of this study, performed in late winter, when the upwelling is strongest, was to describe the development of the bacterial communities in aging upwelled water and to gain insight into the primary drivers of bacterial community composition. To follow successive developments we used an Eulerian approach, investigating water masses from different source areas and of different ages after the upwelling, rather than following the development of a single aging water mass (Langrangian approach). However, Mohrholz et al. (2014) found that most surface water along the transect stems from an upwelling band located along the coast and characterized by similar hydrographic conditions. Our data provide insights into the response of the bacterial community to different developmental stages after the upwelling and to an upwelling-induced phytoplankton bloom. During our study reproducible changes in PA, BPP, and BCC were determined in the two sampled transects. These changes were mainly caused by the abundance and quality of phytoplankton and to some extent resembled the bacterial succession that occurs in temperate water during phytoplankton blooms (e.g., Teeling et al., 2012).

### Phyto-Bacterioplankton Coupling

The measured PA corresponded well to the Chl-a concentration along the transects, while BPP peaked closer to the shore, where nutrient concentrations were high, and nearly reached its minimum during the Chl-a peak. This resulted in a significant correlation between upwelled nutrients (NO_3_, PO_4_) and BPP such that bacterial growth at this location was not carbon-limited but was instead stimulated most likely by the upwelled inorganic nutrients. This conclusion is supported by an estimation of bacterial carbon requirements (data not shown) based on BPP measurements and using a theoretical bacterial growth efficiency of 30%, as previously determined in other upwelling systems (Sorokin and Mikheev, 1979; Lønborg et al., 2011). These calculations suggest that the estimated primary production (Fernández-Urruzola et al., 2014) was sufficient to meet bacterial carbon demands along the entire length of each transect. It is also possible that BPP was stimulated by upwelled refractory dissolved organic carbon (DOC) from the deep-ocean which became more bioavailable due to photochemical transformations after reaching the surface (Benner and Biddanda, 1998). We therefore hypothesize that the initial upwelled bacterial community benefited from upwelled nutrients and photosynthetically derived organic carbon from the dinoflagellates that were dominant in this area (Hansen et al., 2014).

The mean PA is in accordance with the bacterial numbers previously reported from the Benguela upwelling (Brown et al., 1991) and from other upwelling regions (Wiebinga et al., 1997; Barbosa et al., 2001; Cuevas et al., 2004). The low PA in the active upwelling is in accordance with the low cell numbers characteristic of deep water after the cells have been moved to the surface. A strong coupling between PA and the developing phytoplankton bloom after an upwelling has also been reported for the northwest Indian Ocean, the southern Benguela upwelling, and the Chilean upwelling (Painting, 1993a; Wiebinga et al., 1997; Cuevas et al., 2004).

The increase in BPP was comparable to that in previous reports from the northern and southern Benguela upwellings and the Chilean upwelling (McManus and Peterson, 1988; Painting et al., 1989; Brown et al., 1991). Several studies have confirmed the high level of bacterial activity in newly upwelled water (Sorokin and Mikheev, 1979; McManus and Peterson, 1988; de Carvalho and Gonzalez Rodriguez, 2004). Their results are in accordance with the earlier observations of Vinogradov and Shushkina (1978) and Sorokin and Mikheev (1979), who suggested that upwelled water passes through heterotrophic and autotrophic phases as it moves offshore. McManus and Peterson (1988) hypothesized that bacteria in newly upwelled water are stimulated by the sinking substrates generated by herbivory in offshore waters and advected inshore during an upwelling.

The low level of BPP during the peak Chl-a concentration has several possible explanations. Hansen et al. (2014) observed, for the same transect, a significant change in phytoplankton community composition between the upwelling and the Chl-a maximum. Whereas coastal stations were dominated by dinoflagellates, approximately 50 km offshore there was a drastic shift to a diatom-dominated community, which resulted in maximum Chl-a concentrations at a distance of 70 km. Our Eulerian approach prevented an assessment of the developmental stage of the diatom bloom during the Chl-a peak. However, the reduction in BPP might have been linked to the young age of the diatom bloom, when less DOC is produced such that bacteria must attach to phytoplankton cells in order to hydrolyze the dissolved organic matter (Smith et al., 1995). Furthermore, the high abundance of HNF (data not shown) and their marginally significant correlation with bacterial abundance would have led to a predominant reduction of active cells by grazing (Jürgens and Matz, 2002).

In contrast to our observations, McManus and Peterson (1988) and Painting (1993b) reported an increase in bacterial production during and after the peak of primary production. This discrepancy might reflect the fact that we did not sample the diatom post-bloom and therefore missed a second peak in BPP. However, phytoplankton, bacteria, and the control of the latter by bacterivores are closely related, as shown in simulations of different biomass relationships in plankton communities after an upwelling (Moloney and Field, 1991). Sarmento and Gasol (2012) were therefore able to show that DOC originating from different phytoplankton species differentially stimulated heterotrophic prokaryotes. Furthermore, an increase in BPP during the diatom post-bloom might have been hindered by the dynamic conditions in this area, with potentially high HNF grazing and sinking of diatom cells. This conclusion is supported by the rapid decrease in PA 90 km offshore, probably due to predation by bacterivores, as demonstrated in a microcosm simulation of the upwelling in the southern Benguela (Painting et al., 1989).

### Zonation of Bacterioplankton Communities

The influence of phytoplankton development on PA and BPP was even more obvious when the shifts in BCC were considered. The dominance of the taxonomic groups *Alphaproteobacteria* clade SAR11, *Cyanobacteria* clade GpIIa, *Gammaproteobacteria*, *Bacteroidetes*, and *Verrucomicrobia* was in accordance with previous studies in the eastern Atlantic Ocean (Alonso-Sáez and Arístegui, 2007; Friedline et al., 2012) and indicative of a large-scale stable community structure. However, the upwelling induced spatial variability in the structure of the bacterial assemblage, as suggested by other studies of upwelling systems (Baltar et al., 2007; Alonso-Gutiérrez et al., 2009; Teira et al., 2009b; Zeigler Allen et al., 2012).

Parallels in abundance patterns were identified using k-means clustering of the 25 most abundant OTUs, assuming that similar distributions along the transect indicated similar lifestyles. Five distinct clusters were present in both transects, consistent with the strong effects of the presence and absence of phytoplankton, possibly related to copiotrophic or oligotrophic lifestyles. Interestingly, most of the phyla were exclusively represented in either a positively or a negatively correlated cluster, which suggests that different taxonomic groups are influenced by different growth-controlling factors (Gasol et al., 2008).

A PCoA of OTU abundance revealed four clusters of microbial assemblages along the transects. A correlation analysis showed that Chl-a concentrations had a significant impact on zonation as the driver separating the microbial assemblages. This finding was consistent with the observed spatial changes in BCC that are typical for the different temporal stages of phytoplankton bloom development along coastal waters (e.g., Gilbert et al., 2012; Teeling et al., 2012). The characteristics of the four zones were as follows:

Zone 1 comprised freshly upwelled water with low Chl-a concentrations, the dominance of dinoflagellates, high nutrient concentrations, and a low PA. Both the high Shannon index and the high richness indicated the high microbial diversity in this zone, which was probably caused by the mixing of upwelled bacteria from deeper waters with the coastal surface community. This conclusion is supported by the high relative abundance of an OTU with high similarity to sequences derived from oxygen minimum zones and affiliated with *Oceanospirillalespa*. These sequences were reported to be common in the mesopelagic zone and in the dark ocean (Swan et al., 2011; Friedline et al., 2012). The high abundance of other *Gammaproteobacteria* and *Bacteroidetes* in the upwelling zone was in agreement with the findings of Baltar et al. (2007) in their study of a coastal transition zone and indicated the stimulation of *Gammaproteobacteria* by upwelled nutrients (Eilers et al., 2000; Alonso-Sáez and Arístegui, 2007).

Zone 2 was characterized by blooming phytoplankton; however, it could be divided into a section with high abundance of dinoflagellates and high PA and production, all of which occurred at stations with lower Chl-a concentrations, and a second section characterized by the dominance of diatoms, a high PA, and low levels of BPP, occurring at stations with high Chl-a concentrations (Hansen et al., 2014). We found both a high diversity and a high relative abundance of OTUs affiliated with *Bacteroidetes* class *Flavobacteria*, the abundance of which is known to increase during phytoplankton blooms (Teeling et al., 2012; Williams et al., 2012), especially of diatoms (Pinhassi et al., 2004; Grossart et al., 2005). These bacteria have been identified as initial degraders of complex organic matter (Kirchman, 2002; Arnosti, 2011; Gómez-Pereira et al., 2012). An abundant OTU affiliated with *Gammaproteobacteria* clade SAR86 was present in the first section of zone 2, consistent with earlier studies in which the SAR86 group was highly abundant at coastal stations of the Benguela upwelling system (Morris et al., 2012). Suzuki et al. (2001) hypothesized that the SAR86 group is stimulated by macronutrients in upwelled water. This is supported by the potential for proteorhodopsin-based ATP generation (Dupont et al., 2012). Zone 2 was also characterized by a dramatic increase in the relative abundance of *Verrucomicrobia*, mainly because of a single OTU affiliated with *Persicirhabdus,* verrucomicrobial subdivision 1, which became the dominant OTU as Chl-a concentrations reached a maximum. *Verrucomicrobia* subdivision 1 is frequently found in marine bacterial communities (Bano and Hollibaugh, 2002; Freitas et al., 2012). Although the type species *Persicirhabdus sediminis* has been isolated (Yoon et al., 2008), little is known about the function of these organisms. Recent studies provided evidence of the high polysaccharide activity of *Verrucomicrobia,* in which polymer degradation may be more efficient than that by members of *Bacteroidetes* (Martinez-Garcia et al., 2012). Friedline et al. (2012) compared bacterial communities along the eastern Atlantic Ocean and found a similar strong shift to a high abundance of *Verrucomicrobia* during a diatom-dominated bloom. Similarly, in our study there was a significant positive correlation between *Persicirhabdus* and Chl-a concentrations.

Zone 3 was characterized by low Chl-a concentrations, a dominance of dinoflagellates, a low PA, low-level BPP, and a bacterial community shift on the phylum level to a dominance of *Alphaproteobacteria*, the largest proportion of which was made up by an OTU affiliated with “Pelagibacterales.” The ecological role of “Pelagibacterales” in the ocean and in the presence of phytoplankton blooms has been studied extensively (e.g., Morris et al., 2002, 2012; Fuchs et al., 2005; Teira et al., 2009a). “Pelagibacterales” dominate in oligotrophic conditions with low Chl-a concentrations and can be stimulated by phytoplankton-derived labile compounds made available by *Flavobacteria* (Williams et al., 2012). This finding is in accordance with our observation of a high abundance of “Pelagibacterales” after the decay of the phytoplankton bloom. The co-occurrence of “Pelagibacterales” with the SAR86 group is consistent with the report of Dupont et al. (2012), who suggested that “Pelagibacterales” and SAR86 are not metabolic generalists and can thus avoid competing for DOC by utilizing different compounds.

Zone 4 was characterized by higher Chl-a concentrations and a dominance of diatoms due to an invading water filament of younger upwelled water, which resulted in higher PA, increased BPP, and a bacterial community more closely related to the community in zone 2 than in zone 3. Consequently, the relative abundances of both *Verrucomicrobia* and *Flavobacteria* increased in zone 4.

Overall, the zonation of the microbial communities along the aging upwelled water in the Benguela system was stable, with the quality and quantity of phytoplankton and nutrients acting as the main drivers of the observed zonation. The spatial shifts in BCC observed in this study were comparable with the temporal succession stages of algal blooms in temperate seas. Thus, the perennial Benguela upwelling system provides ideal conditions for investigations of the mechanisms linking bacteria and phytoplankton in the ocean. For a more comprehensive understanding of the observed bacterial successions, future studies should analyze species-specific patterns in bacterial substrate utilization for an understanding of their functional role, as well as the influence of top-down effects on the bacterial community composition.

## Author Contributions

BB took and processed the samples. BB and KJ designed the sampling scheme. BB and DH analyzed the sequence data. BB wrote the manuscript. DH and KJ did proof-reading of the manuscript.

## Conflict of Interest Statement

The authors declare that the research was conducted in the absence of any commercial or financial relationships that could be construed as a potential conflict of interest.
