# Supplementary material for: Zonation of bacterioplankton communities along aging upwelled water in the northern Benguela upwelling
Source: Front Microbiol. 2015 Jun 18;6:621. doi: 10.3389/fmicb.2015.00621 (PMC4471433; doi:10.3389/fmicb.2015.00621)
Supplement: Supplementary file 1 [file Presentation_1.PDF]

Supplementary Material

Zonation of bacterioplankton communities along aging upwelled water in the northern Benguela upwelling

Benjamin Bergen[1], Daniel P. R. Herlemann[1], Klaus Jürgens[1*]

[1] Leibniz Institute for Baltic Sea Research Warnemünde (IOW), Seestrasse 15, D-18119 Rostock

[1] **Correspondence**:
Klaus Jürgens
Leibniz-Institut für Ostseeforschung
Seestrasse 15
18119 Rostock, Germany
E-Mail: klaus.juergens@io-warnemuende.de

Supplementary methods:

For the phylogenetic overview, representative sequences (400 bp) were aligned and next neighbors searched in ARB SINA (Pruesse et al., 2012). The results were imported into ARB (Ludwig et al., 2004) using LTP 119 as basis (Munoz et al 2011). Neighboring sequences and close related cultivated representative sequences (>1000 bp) were used to calculate a base tree using PHyML (pos_var_ssuref_bacteria as filter). Short sequences from this study were added using the quick add parsimony tool provided in ARB.

Supplementary references:

Ludwig, W., Strunk, O., Westram, R., Richter, L., Meier, H., Yadhukumar et al. (2004) ARB: a software environment for sequence data. *Nucleic Acids Res* **32**: 1363-1371.

Munoz, R., P. Yarza, W. Ludwig, J. Euzéby, R. Amann, K.H. Schleifer, F.O. Glöckner, and R. Rosselló-Móra. (2011). Release LTPs104 of the All-Species Living Tree. *Syst Appl Microbiol* **34**:169-170.

Pruesse, E., Peplies, J. and Glöckner, F.O. (2012) SINA: accurate high-throughput multiple sequence alignment of ribosomal RNA genes. *Bioinformatics*, **28**, 1823-1829
