# Supplementary figures and images for: Zonation of bacterioplankton communities along aging upwelled water in the northern Benguela upwelling
Source: Front Microbiol. 2015 Jun 18;6:621. doi: 10.3389/fmicb.2015.00621 (PMC4471433; doi:10.3389/fmicb.2015.00621)

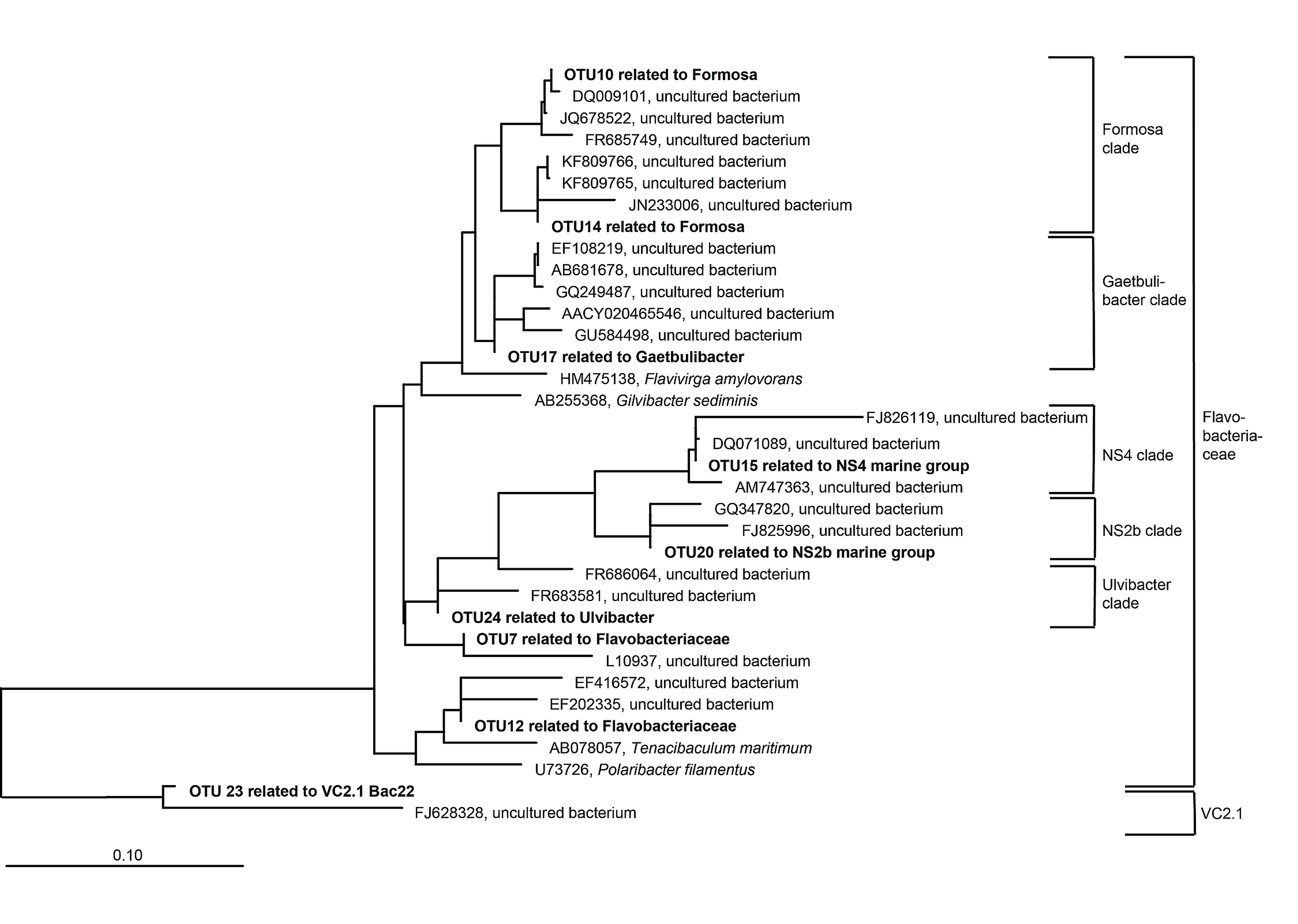

Supplement: Supplementary file 2 [file Image_1.TIF]

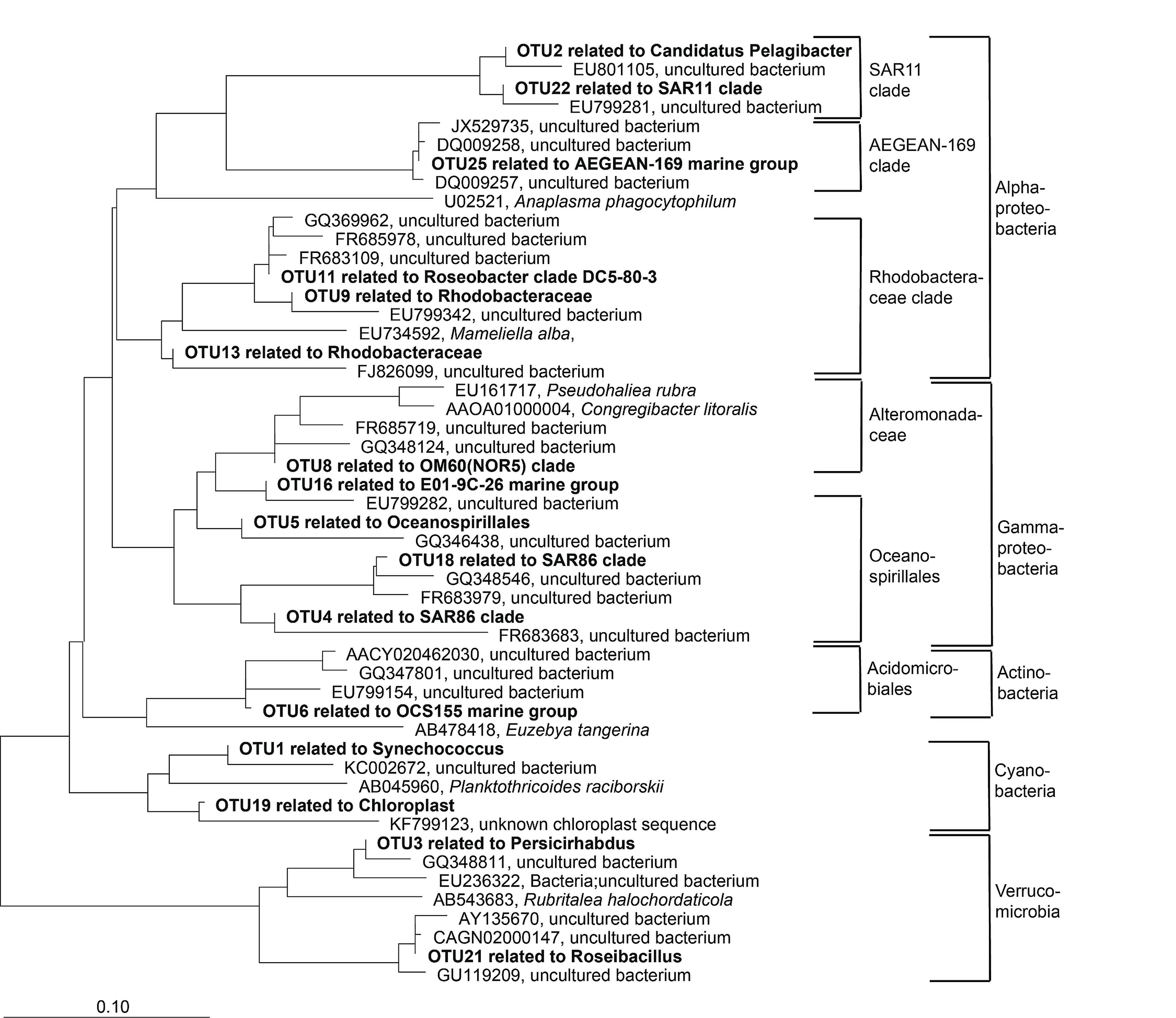

Supplement: Supplementary file 3 [file Image_2.TIF]

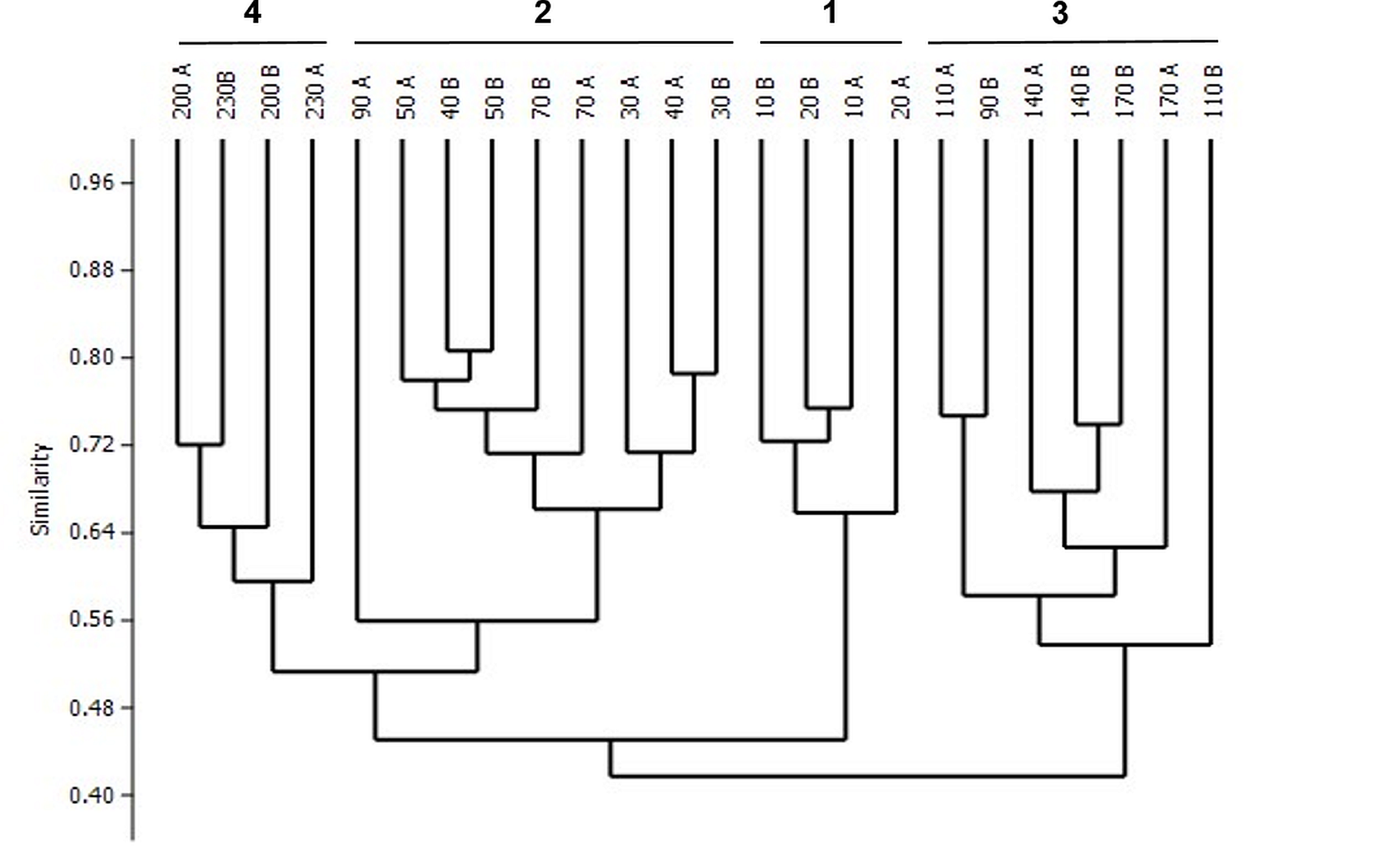

Supplement: Supplementary file 4 [file Image_3.TIF]
